# A Case of Dulaglutide-Induced Vaginal Bleed

**DOI:** 10.7759/cureus.38774

**Published:** 2023-05-09

**Authors:** Christopher J Vaccaro, Syed Muhammad Hussain Zaidi, Peter A Iskander, Erin McFadden

**Affiliations:** 1 College of Medicine, A.T. Still University School of Osteopathic Medicine, Scranton, USA; 2 Internal Medicine, The Wright Center for Graduate Medical Education, Scranton, USA

**Keywords:** diabetes mellitus type 2, fda-approved medications, vaginal bleed, glucagon-like-peptide 1, dulaglutide

## Abstract

Type 2 diabetes mellitus (T2DM) is a growing challenge across the globe. The disease process is amendable to lifestyle modifications in the early stages. If those changes fail to correct endocrine dysfunction, medical therapy is initiated. Initially, therapy for type 2 diabetes consisted of biguanides and sulfonylureas. With modern medicine, we have developed dipeptidyl peptidase-4 inhibitors, sodium-glucose cotransporter-2 inhibitors, and glucagon-like peptide 1 (GLP-1) receptor agonists. Dulaglutide is a GLP-1 receptor agonist that is sold under the brand name Trulicity. The most common side effect associated with Dulaglutide is gastrointestinal discomfort. We present a case of severe vaginal bleeding due to a rare side effect of Dulaglutide.

A 44-year-old perimenopausal female with a past medical history of type 2 diabetes mellitus presented to the clinic after experiencing significant vaginal bleeding. The patient was unable to tolerate Metformin and Semaglutide in the past. The abnormal vaginal hemorrhage started one week after receiving the second dose of Dulaglutide. Her hemoglobin concentration fell significantly. Dulaglutide was immediately discontinued, and her vaginal bleeding stopped.

This case documents the necessity of post-market surveillance to oversee the safety of recently approved medications by the Food and Drug Administration (FDA). Rare side effects can emerge in the general population that were not seen during clinical trials. Physicians should consider the possibility of adverse medication reactions when determining whether to start a new medication or a conventional one.

## Introduction

T2DM is a condition characterized by elevated blood glucose levels due to dysregulation of insulin production or insulin resistance; it is a major contributor to the current obesity epidemic [1]. T2DM affects roughly 422 million people across the globe and about 37.3 million (11.3%) of the total US population [2,3]. It is one of the leading causes of coronary artery disease (CAD), retinopathy, kidney failure, cerebral vascular accidents, myocardial infarction, and lower extremity amputation [2]. The early stages of T2DM are amenable to diet and exercise. Pharmacological intervention is indicated when lifestyle modifications are not sufficient. Glucagon-like peptide 1 (GLP-1) receptor agonists use physiologic mechanisms to lower blood glucose levels and encourage weight loss. GLP-1 receptor agonists are indicated for use in people with type 2 diabetes as monotherapy or in combination therapy with other diabetic medications [4]. GLP-1 receptor agonists work by stimulating glucose-dependent insulin release from pancreatic beta cells. Additionally, this class of drugs has been shown to slow gastric emptying, decrease postprandial glucagon release, and decrease food intake [5]. Providers are encouraged to exercise caution when prescribing any medication in the GLP-1 receptor agonist drug class in patients with a history of multiple endocrine neoplasia 2A or 2B, medullary thyroid cancer, renal impairment, and pancreatitis [6]. Common side effects mostly consist of nausea, vomiting, and diarrhea [7]. Additionally, Dulaglutide carries a black box warning informing of the potential for the development of thyroid c-cell tumors from rat studies [6]. The rare side effects of Dulaglutide have not been fully delineated, as most clinical trials have occurred within the past five years.

## Case presentation

A 44-year-old Caucasian female with a past medical history of essential hypertension, CAD, and hypothyroidism presented to the outpatient clinic regarding newly diagnosed T2DM. Their hemoglobin A1c was 7.6%, and she was started on Metformin 500 mg twice per day. On follow-up appointments, she reported significant nausea and diarrhea from Metformin use. The patient was transitioned to weekly Semaglutide subcutaneous injections. She tolerated the medicine well, but due to market shortages, she was switched to weekly Dulaglutide subcutaneous injections. Within three weeks of treatment on Dulaglutide, she reported having significant vaginal bleeding with clots. She stopped taking Aspirin (81 milligrams per day) upon the onset of bleeding. After bleeding for 28 consecutive days, she became severely fatigued and short of breath, prompting a call to her primary care physician. The physician ordered a complete blood count, placed an urgent gynecology referral, and instructed the patient to schedule an appointment in the clinic as soon as possible. At the same time, her insurance declined a refill of Dulaglutide. While awaiting a refill, the patient ran out of medication, and the vaginal bleeding stopped one week later. In the clinic, she was discovered to be significantly anemic, with a hemoglobin concentration of 7.6 mg/dL (12-16 g/dL). She denied any past medical history of anemia or recent bloody stools. She was not on any anticoagulants. In subsequent visits, the hemoglobin concentration normalized, and no further episodes of vaginal bleeding have occurred since Dulaglutide was discontinued.

## Discussion

Dulaglutide, sold under the brand name Trulicity, is a GLP-1 receptor agonist developed by the pharmaceutical company Eli Lilly in 2014. The drug is administered by subcutaneous injection once per week. It has been shown to lower fasting blood glucose levels, decrease hemoglobin A1c, and reduce body weight [8]. According to the eHealthMe database, to date, there have only been 41 reported cases of abnormal vaginal bleeding out of 59,935 Dulaglutide users [9]. This is a rare side effect that is currently being investigated as part of a Phase IV clinical trial. Healthcare providers should exercise caution when initiating newly approved medications and closely monitor a patient’s response to them. Recently approved medications have passed safety and efficacy tests; however, all adverse effects may not have been fully delineated due to the sample size of the clinical trials. Once the drug is made available to the public, it enters Phase IV of the clinical trial, also called post-market surveillance. These investigations seek information from the public to look for adverse effects that were not observed in smaller populations [10]. Therefore, it is critical for physicians to keep an open and receptive mind to patients’ seemingly unrelated complaints, as an undiscovered side effect could have manifested. During 2020-2022, the FDA recalled 3,933 drugs from the market due to unforeseen adverse effects or the discovery of their potential to do harm [11].

GLP-1 is secreted by ileum L-type cells in response to food intake. GLP-1 is most notable for its ability to stimulate glucose-dependent insulin release from the pancreas [12]. However, the GLP-1 receptor is present in many tissues throughout the body, including the heart, lungs, and central nervous system [13]. The mechanism for vaginal bleeding in response to a GLP-1 receptor agonist is not fully understood. A study demonstrated the effects of GLP-1 administration on female rats. It suggested that “GLP-1 regulates the hypothalamic-pituitary-gonadal (HPG) axis by activating the kisspeptin hypothalamic system, which, in turn, increases GnRH levels as a principal mechanism to stimulate LH secretion to increase gonadal steroid circulating levels” [14]. We hypothesize that Dulaglutide induces vaginal bleeding by causing central endocrine dysfunction.

## Conclusions

This case report documents that severe vaginal bleeding is a rare side effect of Dulaglutide. Providers must examine whether to introduce a newly approved, potentially superior medication as opposed to starting a traditional drug with an established side effect profile. For these critical conversations, we encourage clinicians to have an open and honest dialogue with their patients regarding the risks, benefits, and alternatives before initiating any new medication. In particular, clinicians should discuss the potential for unknown side effects to manifest with newly approved drugs and instruct patients to closely monitor for reactions. Healthcare providers and patients should work together with the FDA to help monitor the long-term safety of newly approved medications.
